# Prevalence of HER2 Positivity and Its Clinicopathological Correlation in Locally Advanced/Metastatic Gastric Cancer Patients in Malaysia

**DOI:** 10.1007/s12029-017-9921-1

**Published:** 2017-01-26

**Authors:** Pathmanathan Rajadurai, Ho Kean Fatt, Foo Yoke Ching

**Affiliations:** 1 Department of Pathology, Subang Jaya Medical Centre, Subang Jaya, Sunway Medical Centre, Monash University Malaysia, 1, Jalan SS 12/1A, 47500 Subang Jaya, Selangor Malaysia; 2 Mount Miriam Cancer Hospital, Tanjung Tokong, Penang Malaysia; 3 Subang Jaya Medical Centre, Subang Jaya, Selangor Malaysia

**Keywords:** Genes, HER2, Gastric cancer, Malaysia, Immunohistochemistry, Gastroesophageal junction cancer, DISH

## Abstract

**Purpose:**

Human epidermal growth factor receptor 2 (*Erbb2*/HER2) overexpression, which was previously detected in invasive breast cancer, has now been implicated in advanced gastric cancer (GC) and gastroesophageal junction cancer (GEC). A study was conducted to determine the rate of HER2 positivity in patients with locally advanced or metastatic GC and GEC in Malaysia and to assess the impact of various demographic and clinical parameters on HER2 positivity.

**Methods:**

A total of 228 adult patients with GC or GEC were enrolled from Subang Jaya Medical Centre, Malaysia, for retrospective (210) and prospective study. All patients were subjected to the HER2 immunohistochemistry test using an FDA-approved, standardized test kit. Carcinomas scoring 2+ on immunohistochemistry were further tested with HER2 in situ hybridization (ISH) using an FDA-approved test kit.

**Results:**

The overall rate of HER2 positivity in the population studied was 24.6% (*n* = 56). The rate was significantly higher in men than in women (29.6 vs. 16.3%; *p* = 0.024). HER2 overexpression was significantly more common in diffuse type than in intestinal type of tumors (39.8 vs. 14.9%; *p* < 0.001). In our study, out of 56 samples, 44 (78.6%) were considered for gene amplification testing, out of which 40 (90.1%) samples showed gene amplification. There was no statistically significant correlation between HER2 positivity and patient age, race, tumor location, tumor differentiation, and TNM staging.

**Conclusions:**

HER2 overexpression was evident in nearly 25% of the Malaysian patients with locally advanced or metastatic gastric cancer. The overexpression correlated significantly with male gender and diffuse-type tumors. The majority of the IHC-positive tumors demonstrated c-*erb2* gene amplification and this finding reached statistical significance.

## Introduction

The International Agency for Research on Cancer (IARC) suggests that gastric cancer (GC) is the fifth most common cancer in the world based on its GLOBOCAN 2012 project data. Gastric cancer is also the third leading cause of cancer mortality in both sexes worldwide (723,000 deaths) [1]. The majority of patients with GC presents at an advanced stage and experiences significant morbidity and mortality [2]. The age-standardized incidence rate (ASR) of GC is about twice as high in men as in women [1].

More than 70% of GC cases are diagnosed in developing countries. More than 727,000 cases of GC were diagnosed in Asia in 2008 accounting for 11.9% of all the cancers diagnosed [3]. Nearly half the total global cases of GC occur in Eastern Asia, mainly in China [1]. The report issued by the National Cancer Registry, Malaysia, in 2006 showed that the total incidence of stomach cancer in the country was 3.9% [4]. However, the incidence of GC varies among people of Chinese, Malay, and Indian origin [5]. Higher rates of GC have been observed in the Chinese population compared to the Malay and Indian populations [6].

Human epidermal growth factor receptor 2 (*erb2*/HER2) is a member of the HER family and is a proto-oncogene encoded by *erb2* on chromosome 17. It plays a major role in promoting cell proliferation and suppressing apoptosis and thus facilitates excessive or uncontrolled cell growth and tumorigenesis. Although HER2 expression was initially associated with breast cancer, it has now been implicated in advanced gastric and gastroesophageal junction cancer (GEC) [7]. While 15–25% of all patients with breast cancer have been found to be HER2 positive, the rate of HER2 positivity varies widely among patients with GC. HER2 positivity has been found to range from 6.8–34% on immunohistochemistry (IHC) and 7.1–42.6% on fluorescent in situ hybridization (FISH) in GC [8].

HER2 is a well-established therapeutic target in breast cancer [2]. Preclinical evidence attests to the significant antitumor efficacy of anti-HER2 therapies (particularly monoclonal antibodies) in GC [9]. Trastuzumab, an anti-HER2 humanized monoclonal antibody [10], already in use for prolonging overall survival and progression-free survival in patients with HER2-positive breast cancer, has now been shown to significantly prolong survival in patients with GC and GEC. Other agents that target HER2, including lapatinib, emtansine (T-DM1), and pertuzumab are also being developed and have demonstrated promising results in HER2-positive breast cancer [7]. The efficacy of lapatinib in combination with capecitabine and oxaliplatin was investigated in a randomized placebo-controlled phase III trial involving 545 patients with HER2-positive advanced gastroesophageal adenocarcinoma. The patients were randomly assigned to capecitabine and oxaliplatin plus lapatinib 1250 mg or placebo daily. The primary endpoint of the study was overall survival. There were no significant differences between the two groups in terms of overall survival although the lapatinib group demonstrated a significantly higher response rate compared to the placebo group [11]. The Trastuzumab for Gastric Cancer (ToGA) trial (a prospective phase III open-label trial) screened 3803 patients with GC/GEC for HER2 status with IHC and FISH test. Patients were considered eligible for the study if their tumor samples were scored as 3+ on IHC or if they were FISH positive (HER2/centromeric probe for chromosome 17 [CEP17] ratio ≥ 2). In this study, 810 patients were found to be HER2 positive. These HER2-positive patients, when treated with trastuzumab in combination with ongoing chemotherapy, showed significant improvement in overall survival as compared to patients who did not receive trastuzumab (13.8 vs. 11. 1 months; hazard ratio 0∙74; 95% CI 0∙60–0∙91; *p* = 0.0046). Post hoc analysis of two large subgroups, one with high HER2 expression (IHC 2+ and FISH positive or IHC 3+; *n* = 446) and the other with low HER2 expression (IHC 0 and FISH positive or IHC 1+ and FISH positive; *n* = 131) was performed. The analysis showed that patients whose tumors had high HER2 expression had a high overall median overall survival (16.0 months) with trastuzumab plus chemotherapy compared to chemotherapy alone (11.8 months) [12]. Thus, HER2 testing has emerged as a promising prognostic marker that could benefit patients with GC/GEC if coupled with an appropriate targeted therapy [2, 7, 10]. Studies conducted so far suggest the need for optimizing HER2 testing as appropriate interpretation of these test results could translate into delivery of optimal therapy. It has been reported that only patients with high levels of HER2 expression derive maximum benefit from trastuzumab therapy [2]. The European Medicines Agency has now recommended that Herceptin (trastuzumab) should be used only in patients with metastatic GC tumors that have HER2 overexpression defined by IHC2+ and a confirmatory ISH+ result, or IHC3+ determined by an accurate and validated assay [13].

A highly varied epidemiological presentation of GC/GEC warrants the conduct of well-designed studies in specific populations/ethnic groups to establish the association between HER2 overexpression and GC treatment. In a recently published study in Japanese patients with GC, tissue expression of HER2 was reported in 6.7% of the 105 patients screened [14].

According to the latest available cancer statistics in Malaysia (2007), 630 cases of GC have been recorded in the country [15]. However, no specific study data has correlated HER2 overexpression with the different stages of GC/GEC in the Malaysian population. A study examining the frequency of HER2 overexpression in GC/GEC in the Malaysian population would therefore provide valuable data unique to Malaysian patients and allow for cost-effective management of the cancer from the standpoint of early diagnosis and optimal therapeutic strategies. Hence, we conducted an observational study to examine the correlation of HER2 overexpression in GC/GEC with parameters such as basic demography, race, pathological subgroups, and site of origin in the Malaysian population.

## Patients and Methods

### Patients

A total of 228 patients with GC/GEC were enrolled from the Subang Jaya Medical Centre, Malaysia, for retrospective (*n* = 210) and prospective study (*n* = 18). The study included men and women over 18 years of age residing in Malaysia, diagnosed with locally advanced, metastatic or recurrent, histopathologically confirmed gastric or GEC. The reasons for exclusion included (i) patients aged <18 years (ii) any other stage of GC/GEC other than that given in the inclusion criteria, and (iii) any other condition or criteria deemed inappropriate by the treating physician for enrollment in the study.

### Study Design

This study was an investigator-initiated, observational study (epidemiological study) wherein the data were collated in both a retrospective and prospective manner.

## Objectives

The primary objective of the study was to determine the incidence of HER2 positivity in patients with locally advanced or metastatic GC and GEC in Malaysia. The secondary objective was to evaluate correlation of HER2 overexpression with demographic and clinicopathological parameters.

### Study Methodology

The pathology records of all patients histopathologically diagnosed with GC/GEC at Subang Jaya Medical Centre (SJMC) were obtained electronically from January 2013 to December 2014 and investigations for HER2 status were included in the study. Among these cases, 210 cases that satisfied the selection criteria were included for the retrospective review. Selection of these retrospective cases was based on the integrity and completeness of the data set. Additionally, 18 patients, who satisfied the criteria for enrollment, were included in the study prospectively. For the prospective enrollment, an open-label, non-randomized, non-interventional design was followed. A cutoff date was set and all patients with GC/GEC who consulted within this cutoff date were selected for the prospective study. Patients who were already receiving treatment for GC/GEC and those who required to be screened for HER status were approached for consent. The approval of the Ethics Committee of SJMC was taken for the study.

### Laboratory Analysis

Tissue samples received from various participating centers were subjected to histopathological examination to confirm the diagnosis of GC and the cases were categorized according to tumor grade and histological subtype.

#### Gene Amplification Testing

Patients with histologically confirmed gastric adenocarcinoma were subjected to HER2 IHC test using an FDA-approved, standardized test kit (Hercep test kit (DakoCytomation Denmark A/S, Glostrup, Denmark). Generally, tumors which were unequivocally confirmed as positive (3+) or negative (0) on IHC were not tested further. However, 30 cases which were 3+ on IHC and all tumors with equivocal results as 2+ on IHC were also tested by dual in situ hybridization (DISH) method to confirm or refute the presence of gene amplification and to explore the presence of ploidy. Dual in situ hybridization was performed using the PathVysion HER2 DNA probe kit (Vysis Inc., Downers Grove, IL) according to the recommendation by the European Medicines Agency (EMA), utilizing the Ventana Ultra platform.

### Study Endpoints

The frequency of HER2 positivity in patients with GC/GEC in Malaysia within the sample size chosen was determined as the primary outcome measure. The secondary outcome measures for the study were as follows:

- Correlation/association of HER2 overexpression with demographic parameters (age, sex, race)
- Correlation/association between HER2 overexpression status and clinicopathological parameters such as (a) tumor location (GC or GEC); (b) tumor subtype (Lauren classification: intestinal, diffuse, or mixed); (c) tumor differentiation (well, moderately, or poorly differentiated); (d) pathological TNM staging (pTNM); and (e) c-*erb*2 gene amplification.

### Statistical Analysis

Data analysis was done using SPSS Software version 20. Demographic characteristics were summarized using descriptive statistics (mean and standard deviation (SD) for continuous variables, and frequency and percentages for categorical variables). Chi-squared test (with Yate’s correction wherever necessary) was used to test the statistical significance of the association of various categorical variables with HER2 status. Continuous variables were grouped for this analysis. A *p* value of <0.05 was considered statistically significant. Higher *p* values indicate higher confidence in rejecting the null hypothesis for no association.

## Results

### Patient Demographics and Tumor Characteristics

The median age of patients was 62 years (range 26–89 years) with the majority being of Chinese (74.1%) followed by Indian origin (7%) (Table 1). Of the 228 cases enrolled in the study, 24.6% (*n* = 56) tested positive for HER2. The location of the tumor was the gastric body in 76.7% of patients. Histological sub typing revealed that 45.2% of the tumors were of the Lauren intestinal type followed by the diffuse type (41.2%); 12.7% of the tumors were classified as “indeterminate” when subtyping was not possible. Poorly differentiated tumors were observed in 125 cases (54.8%) and 42.5% of patients had moderately differentiated tumors. Stage III and IV tumors were observed in 60.1 and 18.9% cases, respectively. Gene (c-*erb*2) amplification was observed in 20.6% of the study population (Table 1).

**Table 1:** Patient demographic and tumor characteristics (*n* = 228)

Variable	Value, *n* (%)
Gender	
M/F ratio	142 (62.2)/86 (37.7)
Ethnicity	
Chinese	169 (74.1)
Indian	16 (7.0)
Malay	15 (6.6)
Others	28 (12.3)
Age (years; [mean ± SD])	60.3 ± 13.7
Age group (years)	
≤30	5 (2.2)
31–45	31 (13.6)
46–59	65 (28.5)
≥ 60	127 (55.7)
HER2 expression	
Negative	172 (75.4)
Positive	56 (24.6)
Tumor location	
Gastric body	175 (76.8)
GEC	53 (23.3)
Tumor subtype	
Intestinal	103 (45.2)
Diffuse	94 (41.2)
Indeterminate	29 (12.7)
Tumor differentiation	
Well differentiated	6 (2.6)
Moderately differentiated	97 (42.5)
Poorly differentiated	125 (54.8)
TNM staging	
II	6 (2.63)
III	137 (60.08)
IV	43 (18.85)
Not known	42 (18.4)
c-ERB-2 gene amplification	
Amplified	47 (20.61)

### Association of HER2 Status with Demographic and Patients Characteristics

A statistically significant correlation was observed between HER2 positivity and male gender. The rate of HER2 positivity was significantly higher in men (29.6%) than women (16.3%) (*p* = 0.024). Notably, 46.7% of the Malay population was tested positive for HER2 compared to 23.7% in the Chinese population although the maximum number of HER2-positive patients was of Chinese origin. The maximum number of HER2-positive tumors was detected in patients aged more than 30 years (Table 2).

**Table 2:** Tumor characteristics by HER2 positivity

Tumor characteristics	HER2-negative *n* (%)	HER2-positive *n* (%)	*p* value
Gender			0.024*
Male	100 (70.4)	42 (29.6)	
Female	72 (83.7)	14 (16.3)	
Age group (years)			0.382
<30	5 (100)	0 (0)	
31–45	22 (71.0)	9 (29.0)	
46–60	53 (81.5)	12 (18.5)	
>60	92 (72.4)	35 (27.6)	
Ethnicity			0.596
Chinese	129 (76.3)	40 (23.7)	
Indian	12 (75)	4 (25)	
Malay	8 (53.3)	7 (46.7)	
Others	23 (82.1)	5 (17.9)	
Tumor location			0.070
Gastric body	137 (78.3)	38 (21.7)	
GEC	35 (66.0)	18 (34.0)	
Tumor subtype			<0.001*
Intestinal	80 (85.1)	14 (14.9)	
Diffuse	62 (60.2)	41 (39.8)	
Indeterminate	29 (100)	0 (0)	
Tumor differentiation			0.063
Well differentiated	3 (50.0)	3 (50.0)	
Moderately differentiated	68 (70.1)	29 (29.9)	
Poorly differentiated	101 (80.8)	24 (19.2)	
TNM staging			0.284
II	5 (83.3)	1 (16.7)	
III	108 (75.8)	29 (24.2)	
IV	29 (67.4)	14 (32.6)	
NK	30 (71.4)	12 (28.6)	

*p < 0.05

### Association/Correlation of HER2 Status with Clinicopathological Features

There was no statistically significant correlation between HER2 positivity and age, race, tumor location, tumor differentiation, and TNM staging. A statistically significant correlation was observed between HER2 positivity and diffuse-type tumor and c-*erb*2 gene amplification.

### Tumor Location

Tumors located in different parts of the stomach such as the fundus, lesser curvature, body, antrum, and pylorus were grouped as gastric body tumors, whereas tumors located in the cardia, esophagus, gastroesophageal junction (GEJ), proximal stomach, and cardio esophageal junction were grouped as GEC tumors. The incidence of HER2 positivity in GEC (34%) was more than that in gastric body (21.7%); however, this association was not statistically significant (*p* = 0.070) (Table 2).

### Histological Grade: Tumor Differentiation

HER2 positivity was not statistically associated (*p* = 0.063) with the histological grade of the tumor; 50% of well differentiated and 30% of moderately differentiated tumors tested positive for HER2 compared with 19% of poorly differentiated tumors (Table 2).

### Tumor Subtype: Lauren Classification

**Fig. 1:**
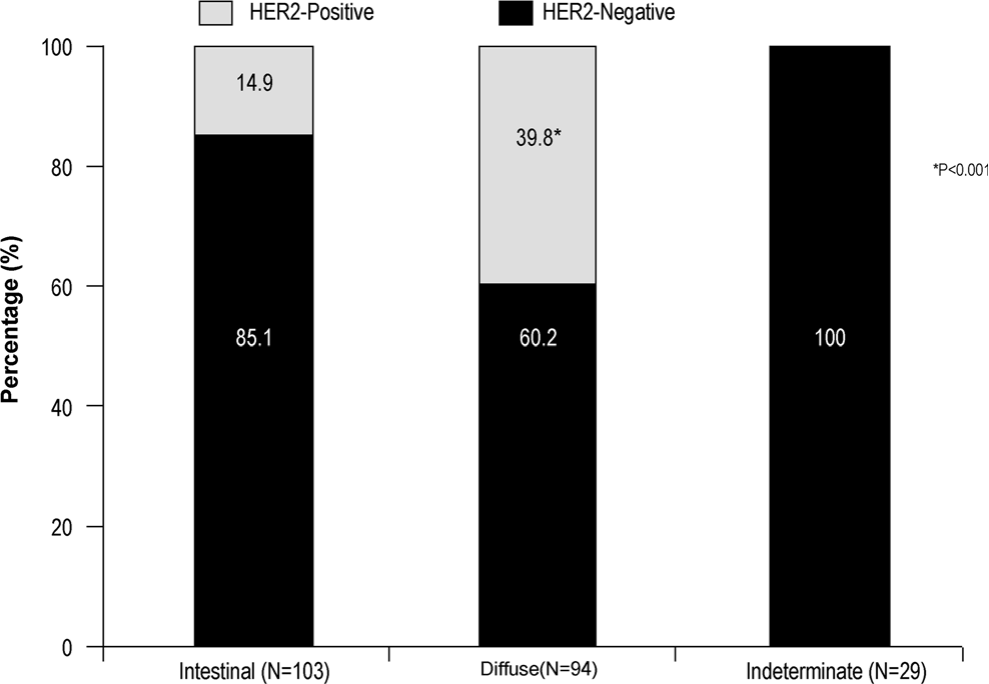
Association of HER2 status with different tumor subtypes

HER2 overexpression was significantly more common (*p* < 0.001) in diffuse-type tumors (39.8%) than intestinal type tumors (14.9%) (Table 2 and Fig. 1).

### Tumor Staging

Overall, 32.6% of stage IV cases tested positive for HER2 as compared to 24.2% of stage III cases (*p* = 0.284) (Table 2).

### Gene (c-*erb*2) Amplification

Only tumors with IHC 2+ (*n* = 16) and 3+ score (*n* = 40) were considered for DISH. Of the 16 cases with IHC 2+ score, DISH was not performed for two tumors as insufficient tumor tissue was available for analysis. Of the remaining 14 tumors, 11 (78.6%) showed gene amplification and 3 tumors did not show gene amplification. Of the 40 cases with IHC 3+ score, DISH was not performed for 10 tumors due to insufficient lesional tissue available for analysis in 6 cases and failure to detect hybridization signals in 4 cases (due to pre-analytical factors). Of the remaining 30 cases, 29 cases (96.7%) showed gene amplification and 1 tumor did not show erb2 gene amplification; this is likely to be a result of tumor heterogeneity for c-erb2 expression, which is a well-recognized phenomenon in gastric cancer. The HER2 positivity on IHC was significantly associated (*p* < 0.001) with gene amplification (Table 3).

**Table 3:** c-*erb*2 gene amplification

No. of samples tested on DISH which are positive on IHC (2+ and 3+) (*n* = 56)	44 (78.6)
Not tested	12 (21.4)
Gene amplification (*n* = 44)	
Amplified	40 (90.1)
Non-amplified	4 (9.9)

## Discussion

The human epidermal growth factor receptors play a central role in the pathogenesis of several human cancers due to their function in regulating cell growth, survival, and differentiation via multiple signal transduction pathways. HER2, which is expressed in many tissues, plays a major role in facilitating uncontrolled cell growth and differentiation. Most studies on HER2 have been carried out in patients with breast cancer. However, thanks to the increasing awareness of the clinical significance of HER2 biology, the role of HER2 in other cancers such as stomach, ovary, uterine serous endometrial carcinoma, colon, bladder, lung, uterine cervix, head and neck, and esophagus cancer has also been identified [16]. This has resulted in rigorous testing of gastric and GEC, notably with a view to improve survival outcomes.

HER2 overexpression in GC using immunohistochemistry (IHC) was first described in 1986 [9]. More than 20% of gastric cancers have demonstrated HER2 overexpression and/or amplification with the percentage increasing to 33% in GEC tumors [10]. In our study, HER2 positivity was evident in 24.6% of patients. Similar HER2 overexpression rates were reported in a Japanese study, where the rate of HER2 overexpression in 200 resected tumors was found to be 23%. In the same study, gene amplification by FISH was identified in 27.1% of the cases [17]. In our study, out of 56 samples, 44 (78.6%) were considered for gene amplification testing, out of which 40 (90.1%) samples showed gene amplification. Tumors with IHC3+ score showed 96.7% correlation with c-ERB-2 gene amplification, and 78.6% tumors with IHC2+ score showed c-ERB-2 gene amplification.

In our study, no statistically significant correlation was observed between HER2 positivity and age, race, tumor location, tumor differentiation, and TNM staging. However, a statistically significant correlation was observed between HER2 positivity and male gender (29.6%; *p* = 0.024), which may be attributable to the higher number of male patients in our study. Furthermore, gastric adenocarcinomas are more common in males [18]. Although statistically non-significant, HER2 expression was more common in Malay patients (46.7%) followed by patients of Indian origin (25%). Furthermore, studies have reported that HER2 expression is more frequent in GEC compared to GC [9]. In our study, the incidence of HER2 positivity was found to be greater in GEC than in gastric body cancers, but this finding did not reach statistical significance. This association has been confirmed by the ToGA study with a large number of patients which demonstrated HER2 positivity in 32 and 18% in GEC and GC, respectively. Several recent studies have demonstrated an association between HER2 expression and tumors with intestinal type histology. The contributing factors for HER2 overexpression in intestinal type GC are quite complex and require extensive investigation. The association between this oncogene and a specific histologic type indicates that certain characteristics may be preferentially expressed together. However, since not all intestinal type tumors are associated with HER2 expression, more than one factor may be involved [9]. However, in our study, HER2 expression was significantly more common in diffuse-type tumors (*p* < 0.001) than in intestinal type tumors.

Our study showed a non-significant association between HER2 expression and well differentiated tumors. Studies have shown both an association and nonassociation between HER2 overexpression and tumor differentiation. This discrepancy may be attributed to varying sample sizes and lower prevalence of HER2 in GC and GEC. Varying methods of evaluation and scoring schemes with different cutoff points before the establishment of standard guidelines may also have contributed [19]. However, no significant association was observed between HER2 expression and TNM staging.

Increasing evidence suggests that HER2 is an important biomarker of GC and GEC. Many studies have evaluated the association of HER2 status and prognosis in patients with GC. The findings have been inconsistent with some studies demonstrating a significantly worse prognosis in patients with HER2 positivity [20, 21] whereas others showing no association between the HER2 status and prognosis [22, 23]. A few studies have demonstrated a longer median overall survival in HER2-positive compared to HER2-negative patients [22, 24]. In a study by Nakajima et al., HER2 overexpression along with nodal metastasis was considered as one of the independent prognostic factors [25]. Hence, the relationship between HER2 status and prognosis in GC remains a subject mired in controversy [7].

In addition to being implicated in the pathogenesis of cancers, HER2 has also been evaluated as a therapeutic target. It has been successfully targeted in both breast cancers and GC/GEC [16]. Trastuzumab was the first HER2-targeted agent which demonstrated significant clinical activity in the advanced GC and GEC settings [26]. In the ToGA trial, addition of trastuzumab to chemotherapy increased the overall survival from 11.1 to 13.8 months (HR = 0.74, 95% CI = 0.60–0.91; *p* = 0.0046). There was also a significant improvement in progression-free survival and response rate with trastuzumab [12]. Unlike trastuzumab, no benefit in terms of overall survival (OS) was evident when bevacizumab was added to a combination of cisplatin and fluoropyrimidine in patients with GC/GEC [27]. Likewise, cetuximab therapy in combination with capecitabine and cisplatin did not achieve the primary endpoint with a median progression-free survival (PFS) of 4.4 months compared to 5.6 months in patients who received capecitabine-cisplatin alone [28]. The REAL-3 study which involved treatment with modified epirubicin/oxaliplatin/capecitabine (EOC) and panitumumab was terminated prematurely because the patients with advanced esophagogastric adenocarcinoma had a statistically significant lower OS [29]. The combination of fluoropyrimidine and platinum-containing chemotherapy, with the addition of trastuzumab remains the standard of care in HER2 positive populations [26]. The introduction of trastuzumab has opened up avenues for the development of anti-HER2 drugs that may be useful in the treatment of HER2-positive gastric cancer [7].

The Malaysian Cancer Statistics 2007 reported a total of 630 GC cases nationwide. A sample size of 228 in our study would therefore represent approximately one third of the population. Hence, the sample size used in our study can be considered an acceptable representation of the Malaysian population with GC/GEC.

## Conclusion

Accurate assessment of HER2 overexpression in GC/GEC in the Malaysian population provides valuable data unique to Malaysian patients, and allows for cost-effective management of the cancer in this population. Nearly 25% of the population demonstrated HER2 overexpression, which significantly correlated with male gender and diffuse-type GC. The rate of HER2 positivity was higher in the Malays than in other races. The sample size was an acceptable representation of the Malaysian population with GC/GEC. However, a larger prospective study will be better posed to endorse or refute the findings of this study. Furthermore, majority of the IHC-positive tumors demonstrated c-*erb*2 gene amplification and this association was statistically significant.
